# Power-Based Non-Intrusive Condition Monitoring for Terminal Device in Smart Grid †

**DOI:** 10.3390/s20133635

**Published:** 2020-06-28

**Authors:** Guoming Zhang, Xiaoyu Ji, Yanjie Li, Wenyuan Xu

**Affiliations:** School of Electrical Engineering, Zhejiang University, Hangzhou 310027, China; realzgm@zju.edu.cn (G.Z.); yanjieli@zju.edu.cn (Y.L.); wyxu@zju.edu.cn (W.X.)

**Keywords:** power sensor, smart grid, condition monitoring, machine learning

## Abstract

As a critical component in the smart grid, the Distribution Terminal Unit (DTU) dynamically adjusts the running status of the entire smart grid based on the collected electrical parameters to ensure the safe and stable operation of the smart grid. However, as a real-time embedded device, DTU has not only resource constraints but also specific requirements on real-time performance, thus, the traditional anomaly detection method cannot be deployed. To detect the tamper of the program running on DTU, we proposed a power-based non-intrusive condition monitoring method that collects and analyzes the power consumption of DTU using power sensors and machine learning (ML) techniques, the feasibility of this approach is that the power consumption is closely related to the executing code in CPUs, that is when the execution code is tampered with, the power consumption changes accordingly. To validate this idea, we set up a testbed based on DTU and simulated four types of imperceptible attacks that change the code running in ARM and DSP processors, respectively. We generate representative features and select lightweight ML algorithms to detect these attacks. We finally implemented the detection system on the windows and ubuntu platform and validated its effectiveness. The results show that the detection accuracy is up to 99.98% in a non-intrusive and lightweight way.

## 1. Introduction

**Figure 1:**
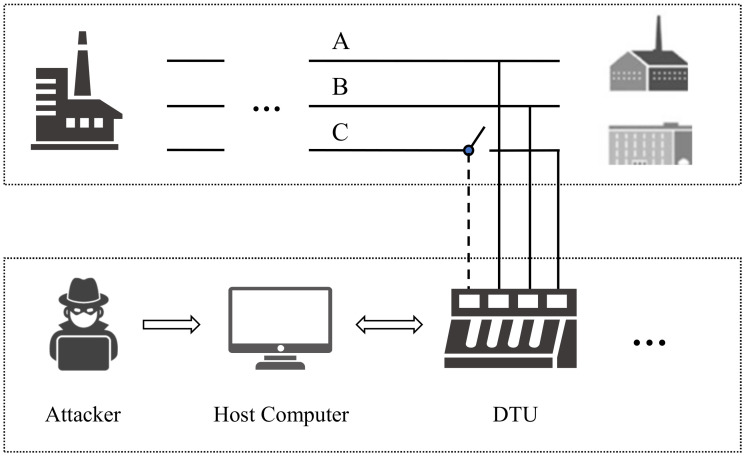
Various security issues have been introduced due to the excessive number of terminal devices in the smart grid.

With the rapid development of smart grids, information and communication technologies are widely applied to smart grids, which makes it more complex in structure and inevitably introduces more security issues. The cyberattack on the Ukraine power grid is a typical smart grid attack event that utilized BlackEnergy version 3 to make seven 110 and 2335 kV substations disconnected for three hours [1]. In 2010, at least 14 industrial sites in Iran were infiltrated by the Stuxnet worm [2], Stuxnet falsified the programmable logic controllers’ (PLCs) programming, resulting in the damaging of centrifuges. Due to the limited resources of those terminal devices, the traditional security detection method cannot be deployed, making it difficult to detect if the device has been compromised. As a critical data collection, communication, and control unit in the smart grid, as shown in Figure 1, once the distribution terminal unit (DTU) is attacked and does not detect in time, it will cause undesirable and often severe economic losses and may even cause major casualties.

The existing protection measures of power industrial control systems include program-based, protocol-based, device-based, and rule-based [3]. For the program-based method, the firmware integrity check tool and malware identification tool have been used for abnormal detection in the corporate environment [4]. However, these technologies are difficult to apply to industrial scenarios. First, these tools are difficult to deploy on legacy devices. Second, the deployment of these tools will affect the original system, so that they may occupy the constrained resources. Moreover, if an attacker has control over the device, these tools are easy to fail.

Prior work [5] has shown that the malicious code execution of PLC can be identified through the controller’s electromagnetic (EM) emanation signals collected from a proprietary chip. EDDIE [6] also proposed an EM-based detection of deviations on IoT devices, EDDIE relies on spikes in the EM spectrum that are produced as a result of periodic activity in the monitored execution. However, those EM-based anomaly detection methods are susceptible to electromagnetic interference (EMI) and need to install the electromagnetic probe near the key chip inside the equipment, which may affect the heat dissipation and bring security issues. The most related works are the power-based anomaly detection methods [7,8,9,10,11], which utilize the power consumption related to the running program to detect anomalies of software-defined radios (SDRs), PLC, etc. Narasimhan et al. proposed a new Trojan detection method based on non-invasive, multi-parameter side-channel analysis, which filters noise through dynamic current and proposed a vector generation method and several design/test technologies to improve detection sensitivity [12]. Huang et al. proposed that the decomposition of current waveform into active and nonactive current can improve the identification of equipment in the non-invasive appliance load monitoring, but this method is aimed at coarse-grained device power consumption monitoring [13]. Kim et al. put forward a power-aware malware detection framework for mobile devices. By collecting power samples and building a power consumption history, their method could realize a 99% true positive rate in classifying mobile malware that runs out of battery power [14]. However, their approach applies only to a particular malicious application. Clark et al. proposed an add-on monitoring system, WattsUpDoc, which could detect malware on medical devices through power side-channel [15]. However, the detection accuracy of previously known malware is only 94%, and that of unknown malware is only 85%. Another significant difference is that our method can monitor the running state of devices containing different CPUs; that is, the change in the program running on each type of CPU can be monitored. This suggests the feasibility of monitoring multi-core CPU or multi-type CPU devices.

There are also some related works to infer the privacy of users using side-channel information and machine learning algorithms. Yan et al. demonstrate that power consumption traces can be used as a practical side channel to gain various types of confidential information of mobile apps running on smartphones [16]. By monitoring the public USB charging, Yang et al. realized that the conjecture of the mobile web page can be viewed with an accuracy of 90% [17]. Conti et al. could distinguish the user’s notebook PC by capturing the smart meter data of the charging station, with an accuracy of 80% [18]. Fan et al. attacked the AES module through correlation power analysis [19]. Although the methods are similar to our work, the above works aim to use power consumption to get the user’s private information.

In recent years, machine learning has made remarkable achievements in computer vision, speech recognition, natural language processing, medical data analysis, and so on, showing the powerful ability of machine learning in solving classification, prediction, and auxiliary decision making. Machine learning technology provides a new way to solve the problem of network security which is difficult to model by traditional methods.

In the field of machine learning, it is divided into supervised learning and unsupervised learning according to whether there are labels in the data set. In supervised learning, each set of data has a label, for example, each piece of data in spam is divided into spam or normal mail. Common supervised learning algorithms include logical regression, artificial neural network, support vector machine, decision tree, random forest, and so on. The data does not contain label information in unsupervised learning, but the intrinsic association of data can be inferred by unsupervised learning algorithms, such as the K-means algorithm, KNN algorithm, hierarchical clustering algorithm, graph clustering algorithm and so on. In our work, we use supervised learning to solve the anomaly detection for terminals of the smart grid. In addition, deep learning, transfer learning, deep enhancement learning, and generative adversarial networks have provided new options for solving intrusion detection. Depth learning is used to solve security problems such as anomaly protocol detection [20], malware detection [21], network intrusion detection [22], BGP anomaly routing detection [23] and differential privacy protection [24]. Transfer learning, which is good at scene or domain transfer, is also used to solve network security problems, such as using transfer learning to correct side-channel signals in hardware Trojan detection [14].

**Figure 2:**
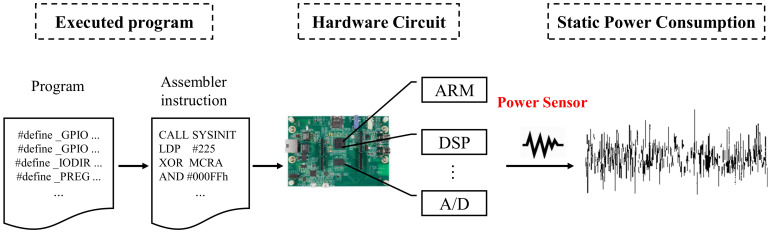
The correlation between the executed program and power signal from the perspective of the Physical Principle. The instructions flip the transistors in the DSP, ARM processor, and other chips gate circuits, resulting in the change of static power consumption.

In this paper, we propose a power-based non-intrusive anomaly detection method to detect the executing program change of DTU using a machine learning method without introducing security issues and affecting the real-time performance of the system. The basic idea is to analyze the power consumption by means of machine learning (ML), and infer whether the applications running on the DTU has changed. As shown in Figure 2, the underlying physical principle is that the DTU always executes fixed applications that do not always change in the whole life cycle, and meanwhile, it’s hardware characteristics enable a strong corresponding relationship between its power consumption and it’s executing programs. We also found that when the program has been falsified, even the behavior of the DTU looks normal, the power consumption will also change accordingly. Therefore, by analyzing the real-time operating power, it is possible to detect the running state of the DTU.

Note that as the DTUs run on VxWorks, which is a real-time operating system (RTOS), in order to increase the character of real-time, DTU adopts two different CPU units (including a DSP and ARM processor), thus, all the jobs (such as communication, display, FFT, etc.) can be processed in real-time with the distributing calculation of the two CPUs that are in charge of different applications. For example, the ARM processor is responsible for communication and switch control applications, DSP runs the applications of data collection and processing. Due to the fact that the ARM and DSP are integrated on the same chip, it hard to extract the corresponding power traces of each CPU to detect program change respectively. In this paper, we obtain the power of two CPUs and its peripheral components by measure the voltage drop across a resistor that connects between the motherboard and DC power supply, as the voltage drop is proportional to the power consumption of the target circuit [25].

To validate the idea and implement the anomaly detection approach, we first set up a testbed based on DTU in the laboratory. Secondly, we design a normal program and four attack programs on the DTU, which aims to change the applications separately without being noticed by the operator. Third, we design a power sensor to collect the power traces and chose representative features to capture the difference of power traces. We then use those features to build ML models for anomaly detection. We compare three different types of classifiers: One-class, binary-class, multiclass classification algorithm, and select the best classifiers among seven algorithms. Finally, we implement the scheme on ubuntu.

We show that the anomaly detection scheme achieves a detection accuracy above 99.98% with lower system overhead. In addition, our scheme has the following advantages: the scheme does not require modifying the hardware or software, which does not bring potential vulnerability, and the deployment will not interrupt the normal operation of the power grid. In summary, our contributions are summarized as follows:

- We propose a lightweight and non-intrusive anomaly detection approach on DTU by analyzing the power consumption.
- We explore the strong corresponding relationship between power and applications and design the detection system that can detect the application change by analyzing the power.
- We implement the detection system on Ubuntu and evaluate its performance on the five programs with different ML algorithms, a video demo of the real-time anomaly detection system is available at https://youtu.be/_2GjTkbYWk8. The result shows that the detection system can achieve a detection accuracy of up to 99.98%.

## 2. Materials and Threat Model

### 2.1. Materials

In this work, the resistors were purchased from Shanghai Yixin Trading Co., Ltd. (Shanghai, China). The DTU and power supply were purchased from CYG Sunri Co., Ltd. (Shenzhen, China). The DAQ (DAQ U2541a) were purchased from Keysight Technologies (Santa Rosa, CA, USA).

### 2.2. Threat Model

In this section, we briefly introduce the threat model of the DTU system. The adversary’s goal is to hack the power grid system including DTUs and execute unauthenticated applications that cannot be detected by the traditional security method. We assume that the adversary has the ability to access the DTU by utilizing the vulnerability of the smart grid system or the adversary is the worker who can directly operate and download programs to the DTU, and he actually has been bought to damage the smart grid system.

In this paper, we design four types of attacks against the DTU, which mainly falsify the code running in the DSP and ARM processor. These attacks are characterized by high concealment as the attacked smart device seems to work normally, but the data collected from the smart grid is actually forged. Next, we will introduce the four attacks in detail.

(1) The Switch on/off Attack (Attack1).

The switch on/off attack mainly changes the code running in the ARM processor. One of the most important applications for DTU is to measure various values on the power lines through current or voltage sensors. When some measured values are greater than the preset threshold, the DTU will close the overloaded line through the controlling switch. The switch on/off attack makes the power switch controlled by DTU continuously switch on and switch off at a very fast rate, thereby affecting the normal operation and the stability of the power grid and reducing the service life of the power switch. When the frequency of the switch on/off is high enough, the friction loss of the switch will become very large, and people will not be able to detect the abnormal situation of the power grid.

(2) The Data Collection Attack (Attack2).

The data collection Attack mainly changes the code running in the DSP processor. In general, the sampling rate of the current/voltage collection is about 1000 SPS (Sample Per Second). The attack significantly decreases the sampling rate to 50 SPS, but the frequency of data uploaded to the monitoring center does not change. The lacking samples will be forged according to the collected sample. This type of attack makes DTU unable to accurately and timely sense the status of the smart grid.

(3) The Channel Attack (Attack3).

The channel attack mainly changes the code running in the DSP processor. As we know, the DTU has the ability to monitor the status of several power lines, such as the values of the current and voltage. The channel attack aims to reduce the number of monitoring power lines to one, the current and voltage values of other power lines are forged according to the values of the monitoring line. Therefore, the forged value is difficult to be seen through, and when the unmonitored line is overloaded, the DTU cannot take protection operations based on the actual value, which will bring catastrophic consequences for the smart grid.

(4) The Overload Attack (Attack4).

The overload attack mainly changes the code running in the ARM processor. In addition to running legitimate programs, the attackers will download and run an additional program on DTU that is meaningless but takes up a lot of computing and storage resources, so that the overload attack could deprive the resources of legitimate applications, thus, the legitimate service cannot get a timely response. For example, when the load of the power grid is short-circuited, the DTU cannot measure the current and disconnected the load in time when under the overload attack.

Essentially, these four types of attacks can be regarded as falsification of executed programs inside the device. Note that the operating system version of the Data Collection Attack is V3.30, and the others are V3.10. No matter what operating system version, our proposed attack detection method can defend against those kinds of attacks.

## 3. Methods

In this section, we first introduce the background of power consumption and present the feasibility from the perspective of physical principle and experimental evidence, and then we introduce the design of the monitoring system based on the power consumption side-channel.

### 3.1. Power Side-Channel

Side-channel information refers to the information of sound, power consumption, electromagnetic radiation, and even the running time generated by electronic equipment when the program is running. These channels are different from the channels used in normal communication, so they are called side-channel information. Side-channel signals reflect the execution of a set of particular instructions inside the processor; thus, they can be leveraged to monitor program execution [26].

The power and EM side-channel signals are the most commonly used signals for anomaly detection. In theory, compared with EM signals, the accuracy of using power consumption for anomaly detection is higher because the voltage fluctuation in power consumption caused by code execution will radiate out in the form of EM wave through the components of the circuit, such as the processor or random-access memory, which can act as antennas [26]. And in the process of radiation and reception, the information related to program changes will further be lost. Thus, we select the power consumption signal as the candidate signal.

Another advantage of anomaly detection through power side-channels is that the specific source code running in DTU is not required, or even that we do not need to know which functions the program calls. Traditional malware detection requires code analysis of the program, which is not feasible in our case because the device manufacturer is unwilling to share its code with the users, a situation that exists in many cases.

### 3.2. Feasibility Analysis

In this section, we present how power consumption is related to the executed program from the perspective of physical principles and experimental evidence.

Firstly, from the perspective of physical principles, the root cause of chip power consumption is the flip of the gate circuit. Different programs have diverse instructions and each instruction calls varied modules in the chip, so the power consumption also should be different. So it is feasible to distinguish different programs by power consumption information from the view of physical principles.

**Figure 3:**
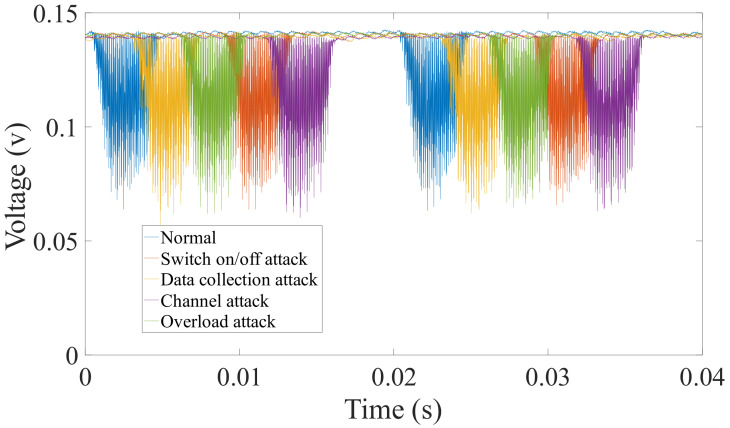
The power traces in the time domain of Distribution Terminal Unit (DTU) under different attacks. The running cycles of each program are the same, but the amplitudes of power are slightly different.

**Figure 4:**
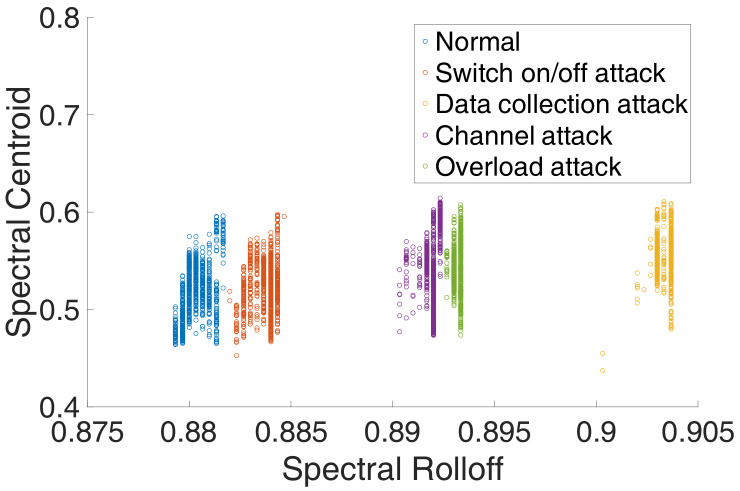
The feasibility to classify different attacks with different features. The features of each type of power signal cluster together separately, because the data collection attack runs in another OS version, its features are far away from others’.

Secondly, from the perspective of experimental evidence, we firstly perform simple experiments on the DTU to verify the feasibility of power side-channel analysis. We launched four attacks and collect the corresponding power signal, and extract 34 features with the PyaudioAnalysis [27]. Figure 3 shows the original power signal captured by our data acquisition card, and Figure 4 shows the features of different power signals. We can see from the original power signal that although the overall difference between normal and abnormal programs is small, there are still some minor differences. From the features of different power signals, we can see that the features are distinguishable and the gap between different features is obvious. Moreover, we find that the features from the data collection attack have significant differences with other features. We guess that it is because the data collection attack was run on a different operating system version.

Thus, based on the above description and discussion, we can utilize the machine learning method to detect different attacks.

### 3.3. Overview of System

**Figure 5:**
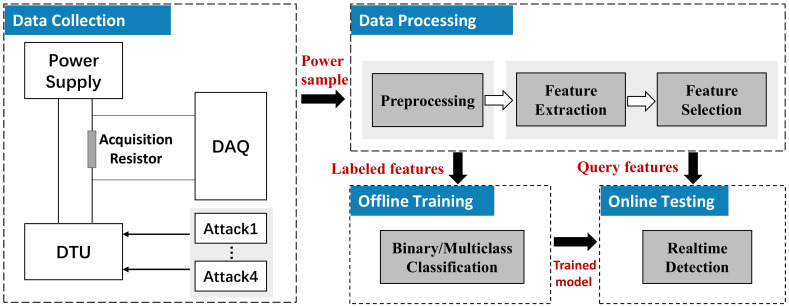
Overview of the real-time monitoring system. It mainly consists of two parts: data collection, and data analysis.

The design of the detection system is mainly composed of two parts, i.e., the hardware design capturing the power consumption of the DTU, and the software design which analyzes the collected power consumption to infer the running status of DTU, as shown in Figure 5. In the following, we will reveal the details of the two parts.

### 3.4. Hardware Design

The key to detecting attacks is to obtain the side-channel information related to the executed program and then extract the relevant features. There are several side-channel collection methods commonly used to obtain the running status of the device, i.e., by capturing the electromagnetic wave and temperature radiation from the devices or collecting the power consumption from the power supply line. Radiation information is vulnerable to environmental interference and will lose some information in the process of radiation and receive, thus, we select the power consumption side-channel information that has strong anti-disturbance ability against the external ambient noise (Algorithm 1).
**Algorithm 1** Power-Based Anomaly Detection.
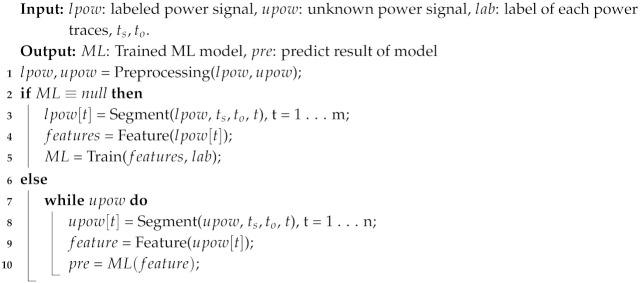


There are two common ways to capture power information, one is to capture the voltage drop of a sampling resistor, and the other utilizes electromagnetic induction from the power supply line. The induction scheme has an advantage over the sampling resistor scheme, that is, it can be completely isolated from the original system, and does not modify the hardware of the original system. However, compared with the resistor scheme, the induction scheme is also susceptible to electromagnetic interference existing everywhere in the environment and also losing valid information. Therefore, we finally chose the sampling resistor as the power sensor.

Specifically, the scheme first collects the power consumption information of DTU by capturing the voltage drop across a resistor, which connected between the power supply and DTU, then transmits the raw power consumption signal to the analysis part, as shown in the left part of Figure 5. Instead of developing a data acquisition instrument [28,29,30,31,32], we use the Agilent U2541A [33] in this paper to capture the voltage drop across the resistor and the Agilent U2541A supports a maximum sampling rate of 250 kHz and 16-bit resolution.

According to our test results, the rated power of the DTU is less than 5 W, and the rated DC voltage is 24 V, thus, the current of the supply power line is below 0.2 A. When the value of the sampling resistor is less than 0.5 Ω, the signal-to-noise ratio (SNR) of collected power traces is low, which cannot accurately reflect the running program in the device. To improve the SNR, we increase the value of the resistor to 1 Ω without affecting the normal operation of DTU. It is also worth mentioning that the power acquisition equipment, power supply module, and sampling resistor need to share the same ground to avoid interference, and at the same time, shielded wires also should be used. Our experiments show that the resistor scheme can retain the information of the original power signal well.

### 3.5. Software Design

When the power consumption signal is transmitted to the computer, the signal needs to be analyzed by binary or multiclass classification to infer whether the executed program has been changed or which program is running. The key challenge of detection is to find the best features combination and choose a lightweight algorithm that can reduce the system resources in the condition of high detection accuracy. The designed algorithm is illustrated in Algorithm 1.

#### 3.5.1. Preprocessing

We first add the low pass filter to remove the DC signal and signals with frequencies below 70 Hz as the problem of power frequency interference is serious. After that, we divide the signal into fragments of the same time length ($t_{s}$), and there is an overlap between adjacent power fragments ($t_{o}$), and last, extract primary features from these power samples.

#### 3.5.2. Feature Extraction and Selection

Feature extraction. We choose as many features as possible to keep useful information from the power traces. In this paper, we use the PyaudioAnalysis [27] to calculating 34 types of features including Zero Crossing Rate, Energy, etc. We then constructed an original 34-dimension vector $\vec{V}$ = {ZCR, Energy, …, Chroma Deviation} for each power consumption trace.

Feature selection. To remove the redundant features that are not that significant and get a better detection performance with less training and testing time, we select the most relevant features using the RandomForest algorithm, which is a supervised machine learning algorithm. Instead of searching for the most important features, the RandomForest searches for the best features in a random subset of features, which leads to a better candidate feature set. So we find a set of basis vectors that can replace the original feature set.

#### 3.5.3. Training and Testing

Training. The binary classification algorithm divides the features of power traces into two categories as normal samples (normal program) and abnormal samples (four types of attacks), thus, the binary classification can only detect whether the executed program has been tampered and cannot detect which attack. In order to identify what kind of tampering the executed program has suffered, we need to use a multiclass classification algorithm in which the testing power trace is divided into exactly one of five classes. In this paper, we adopt the two algorithms to satisfy different requirements.

Testing. In the process of the detection process, we collect the power consumption traces of DTU in real-time and calculate the power features, which will feed into the trained models to detect whether the executed program has been falsified or what kind of attack the device suffered.

## 4. Results

In this section, we verify the effectiveness of the proposed method based on the power consumption side channel. Firstly, we describe the experimental setup and the real-time monitoring system. We also compare the detection performance of three different types of classification algorithms including one-class classification and binary classification and multiclass classification. Then we evaluate the performance and detection accuracy of different ML algorithms. Finally, we evaluate the detection accuracy of different attacks and the influence of different impact factors.

### 4.1. Experimental Setup and Monitoring System

**Figure 6:**
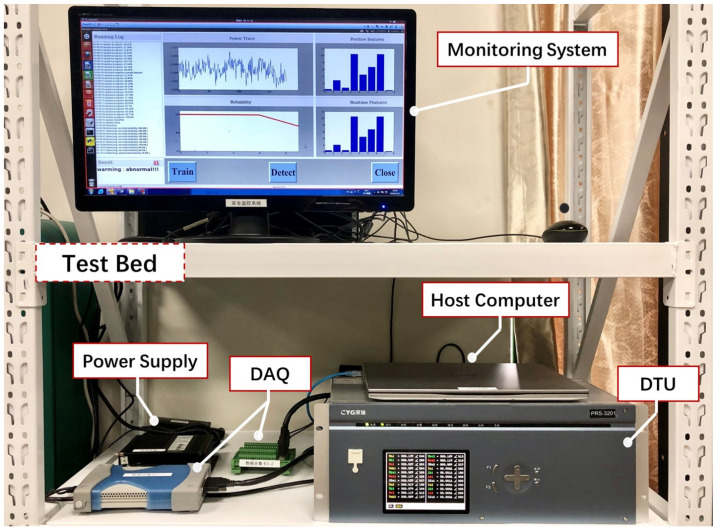
The experimental setup including DTU, host computer, DAQ, power supply, and the monitoring computer.

In our implementation, we collect the raw power consumption on the testbed in our laboratory, as shown in Figure 6. In particular, the resistance of the sampling resistor is 1 Ω, and the sampling rate of the DAQ [33] is up to 250 kSa/s. We collected the power trace for each program with a sampling rate of 60 kSa/s, the total length of the power traces is 50 min. In the following experiments, the collected power consumption is analyzed offline with Python 3.7 for the convenience.

**Figure 7:**
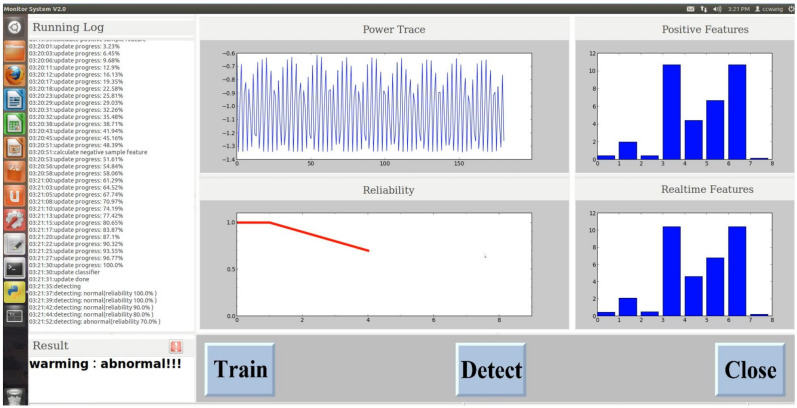
The UI snapshot of the real-time monitoring system.

We also developed a monitoring system based on the proposed method to detect any changes to the running program. The system can run on both Windows and Ubuntu systems. The UI snapshot of the system is shown in Figure 7. The UI mainly contains four parts: (1) The running dialog area which displays the detailed operation information including training and testing process and the test results. (2) The results display area which will show the real-time status, for example, when the system detects that the device has been attacked, this area will show the testing result. (3) The display area of power traces, features, and the confidence level of the normal program running in DTU. (4) Button area including three buttons: Train, Detect, and Close.

### 4.2. Influence of Different Classification Algorithms

There are three types of classification algorithms that can be used for attack detection: One-class classification, binary classification, and multiclass classification. The one-class classification tries to identify objects of a specific class amongst all objects, by primarily learning from a training set containing only the objects of that class [34], thus, it did not need negative samples. Compared with the one-class classification algorithm, the binary classification algorithm requires negative samples for training. These two above algorithms can only identify whether the device has been attacked and cannot identify which attack has been suffered. For multiclass classification algorithm, it can learn from different kinds of attack training sets and then identify which type the attack is.

**Figure 8:**
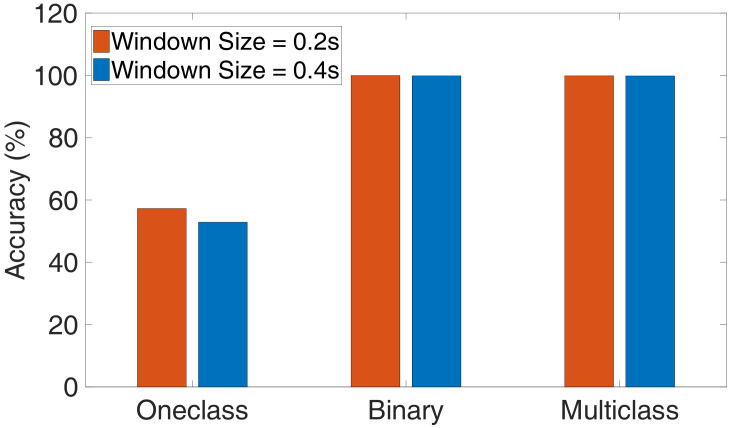
The accuracy of one-class, binary and multiclass classification. The $t_{s}$ and $t_{o}$ are set to 0.4 s, 0.2 s and 0.2 s, 0.1 s.

To evaluate the performance of those algorithms, we choose the Support Vector Machine (SVM) as the classifier, which not only supports one-class, binary classification but also multiclass classification case as well. For one-class classification, we randomly selected 50% power traces of a normal program for training, and the left power traces as the testing set. For binary and multiclass classification, the training set is half of the total data set. We plot the accuracy for each classification algorithm with different sliding window size ($t_{s}$) and overlap time ($t_{o}$, $t_{s}=2t_{o}$) in Figure 8, as we can see that the one-class SVM only has a maximum accuracy of 57.26%, and the binary and multiclass SVM show better detection accuracy, which is up to 99.94% and 99.85%. We can also observe whether $t_{s}$ is 0.2 s or 0.4 s, and the binary SVM has the best performance. Compared with binary SVM, the detection accuracy of multiclass SVM is lower, which is because the binary SVM just needs to classify two different types of samples: normal and abnormal samples (the four types of attacks can be regarded as abnormal programs).

### 4.3. Impact of Different Attacks

Table 1 shows the confusion matrix for the multiclass classification. As we can see, most power traces can be classified correctly. Furthermore, the total traces of Attack2 can be correctly classified, which is because its running OS is different from others. We will further analyze this phenomenon in Section 4.4.

### 4.4. Ability to Detect the Changes to the Executed Program

From Figure 4, we know the features from data collection attack (Attack2) has significant differences to other features. We also guessed that it was because the data collection attack was run on a different operating system version, the executed code in DTU changed the most. To evaluate the ability of how much the executed program changes can be detected, we design the four attacks in which the changes compared to the normal program are different but the fundamental function of those attacks is the same as the normal program, thus, the operator cannot identify the anomaly if the DTU is running those malicious programs. The Attack2 and Attack4 have the most changes, and the attack3 has the least change compared to the normal program.

**Figure 9:**
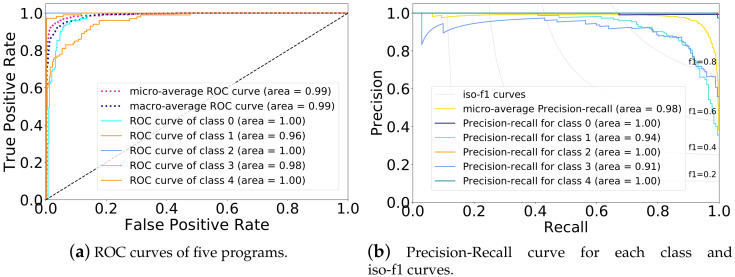
The ROC curves and the Precision-Recall curves of multiclass SVM classification using one-vs-the-rest (OvR) multiclass strategy. Class 0 is the normal program, and class 1 is Attack1, etc. (**a**) ROC curves of five programs; (**b**) Precision-Recall curve for each class and iso-f1 curves.

Figure 9a,b plot the ROC (Receiver Operating Characteristic) curves and Precision-Recall curves for the five programs. We can see that the areas under ROC curves of the normal program, Attack2, and attack4 are 100%, they have the highest detection performance, which means the difference between each of them is the biggest. The detection performance for Attack1 and Attack3 is slightly worse as their program changed the least. According to the above results, we can conclude that there has a strong correlation between power consumption and running programs. Once the device is attacked, its internal operating procedures will be falsified, resulting in changes in power consumption. Even if part of the executed program has been tampered with, we can achieve high-accuracy detection by analyzing power consumption. In fact, the proposed method is to detect the changes in the running program. No matter what kind of attack, as long as the program has tampered with a certain amount, this method can detect it. In this paper, we verify the effectiveness of the algorithm on the four typical application falsifications. In future work, we will systematically explore how many program changes can be detected, and the detection performance on unknown attacks.

As we know, the features of power traces could determine the upper limit of ML detection accuracy and classifiers just approximate this upper limit. However, the chosen classifiers are also very important, because the performance of different classifiers will be varied a lot, and especially, the detection time is also an important indicator in practical application. To evaluate the performance of the classifiers for different attacks, we chose seven commonly used ML algorithms, which are shown in Table 2. The experiments results are shown in Table 2. Firstly, the accuracy of all the algorithms is above 99.7%, and the accuracy of the GradientBoosting and DecisionTree classifier is up to 99.98%. The DecisionTree takes the least running time, so it has the best performance in terms of detection accuracy and running time. Secondly, all the algorithms have the highest detection accuracy for Attack2 and Attack4, which are in agreement with the results in Section 4.3.

### 4.5. The Influence of Features.

The features of power traces have a huge impact on detection performance, the irrelevant features will negatively influence the performance of the ML algorithms and lower the speed of the whole detection process.

**Figure 10:**
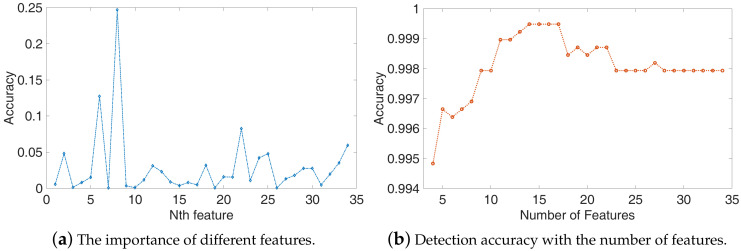
The impact of features on the performance of detection. (**a**) The importance of different features, we use the RandomForest classifier to evaluate the importance of different features; (**b**) Detection accuracy with the top N features.

In our experiment, we use the pyAudioAnalysis to extract 34 features as shown in Table 3, and use the RandomForest classifier to evaluate the importance of features. We plot the importance of features in Figure 10a, and from the results, we can observe that the importance of different features varies greatly and Spectral Rolloff is the most important feature.

Then, we selected the top N features as the feature set and train the SVM algorithm. The accuracy of testing is depicted in Figure 10b, the accuracy increased with the number of features, and when the number of features is 14, the accuracy is up to the maximum accuracy of 99.94%. However, as the number of features increases, the accuracy begins to decrease. In general, more features mean that more information can be extracted from the sample, but only valid features have a good impact on classification. The top 14 important features consist of the most effective feature set, the other features are redundant features which will have a negative influence on the detection performance. So, the redundant features should be removed before the training model and detection.

### 4.6. Influence of Sliding Window Size.

**Figure 11:**
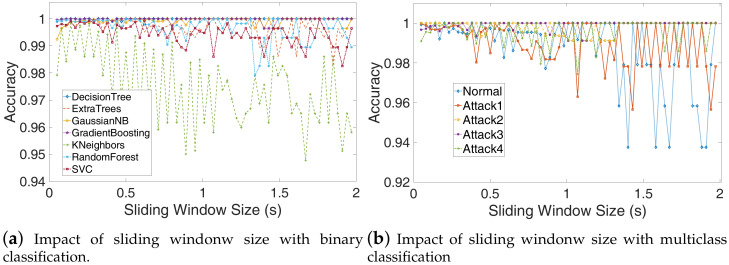
The influence of sliding window size $t_{s}$, the overlap time $t_{o}$ is set to $t_{s}/2$. (**a**) The detection accuracy decreases as window size increase with binary or multiclass SVM, when the window size is about 0.2 s, the algorithms have better performance; (**b**) The detection accuracy of the normal program is lower than the other attacks, which means some normal samples have been classified as other samples but the other attacks sample was not easy to misclassify.

**Figure 12:**
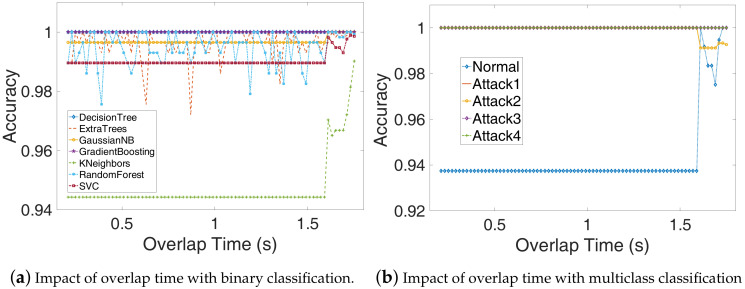
The influence of overlap time $t_{o}$. The sliding window size $t_{s}$ is fixed to 1.8 s. (**a**) The detection accuracy increases as the overlap time increases, this is mainly because when the overlap time is close to window size, the relationship between adjacent samples becomes closer, and each type of sample can better reflect the corresponding running state of the DTU; (**b**) The detection accuracy also increases as the overlap time increases when using the multiclass classification.

The sliding window size also has an important impact on detection accuracy. To select a proper sliding window size, we use the binary and multiclass classification to evaluate the sliding window size ($t_{s}$), which ranges from 0.05 s to 1.97 s, the step is 0.03 s. The overlap time is set to $t_{s}/2$. The evaluation results depicted in Figure 11a,b show that the detection accuracy decreases as window size increase with binary or multiclass SVM, and when the window size is about 0.2s, which approximately equal to 10 times of the execution cycle, the algorithms have better performance. From Figure 11b, we can see that the detection accuracy of the normal program is lower than the other attacks, which means some normal samples have been classified as other samples but the other attacks sample was not easy to misclassify. That is because those attacking programs are modified from the normal program, so the normal program is more similar to every attacking program. We can also see similar results, as shown in Figure 12.

### 4.7. Influence of Overlap Time.

In order to maintain the correlation between adjacent power traces, each pair of them has an overlap. In our experiments, the overlap time ranges from 0.21 s to 1.75 s, the step is 0.02 s, and the window size is fixed to 1.8 s. As shown in Figure 12a,b, the detection accuracy increases as the overlap time increases when the overlap time is greater than 1.61 s, the accuracy increased to above 96.5% for KNeighbors classifier and the detection accuracy of the normal program is also dramatically increased to above 97.5%. This is mainly because when the overlap time is close to window size, the relationship between adjacent samples becomes closer, and each type of sample can better reflect the corresponding running state of the DTU. However, when the overlap time becomes larger, the feature extraction and detection time for a period of power traces will become larger. Thus, the overlap time can be chosen by considering both detection accuracy and detection efficiency. In order to maintain high detection accuracy, the overlap time can be set to 1.61 s when the window size is 1.8 s.

## 5. Discussion

The results presented in this work demonstrate the feasibility of using power consumption to detect the anomaly application running in the terminal devices in a non-intrusive and lightweight way. The results show the close relationship between the running programs and power consumption. That is, the power consumption changed with the changing program of DTU, therefore, the power consumption can be analyzed through machine learning to realize the detection of attacks without introducing security issues and affecting the real-time performance of the system compared to the traditional methods.

Zeus [5] presents a contactless, passive, and non-intrusive control flow integrity monitoring solution for PLCs. Zeus identifies malicious code execution through side-channel analyses of the controller’s electromagnetic emanation signals. The programs used for classification fall in the classes of vector arithmetic, numerical methods, control algorithms, cryptography, signal processing, and communications. As we can see, these programs have totally different functions, which can be identified by the operator when these programs are running, and because of the great difference of program function, the power consumption will also vary greatly. Compared to our method, Zeus did not show the detection performance of the attacks in which the function is the same as the normal programs. Haider et al. propose a malware detection system for critical embedded and cyber-physical systems (CPS) by exploiting electromagnetic (EM) side-channel signals from the device to detect malicious activity [35]. They separately evaluate the system with different malware behavior on different applications using an Altera Nios-II soft-processor and an A13-OLinuXino—a Cortex A8 ARM processor. Compared to our work, they did not show the detection performance on multiprocessors. RFDIDS [36] proposed an air-gapped distributed intrusion detection system that monitors the substation activities by radio frequency (RF) measurements to verify the correctness of the SCADA network traffic. Compared to our approach, they monitor network traffic. Liu et al. proposed a low-cost code execution tracking method, which can monitor the code changes of embedded devices through the power side channel. He builds a hidden Markov model to model the power data of instruction execution and recovered the most likely instruction sequence with the modified Viterbi algorithm [14]. However, their method was aimed at the embedded MCU chip with simple 8051 architecture, while the smart grid terminal equipment is much more complex both in hardware and software.

Although there are important discoveries revealed by these studies, there are also limitations. First, the proposed method can detect whether the running program is a normal or malicious one, but we do not know if it can detect the injection process of malicious code. Second, the detection performance of the anomaly detection model against unknown attacks also needs to be explored. Third, due to the fluctuation of the grid voltage, the output voltage of the DC power supply will also change slightly; thus, the power consumption of the terminal device also changes accordingly, and the future work has to explore the performance of the long-time detection.

In summary, the studies in this paper thus offer a new strategy to detect the anomaly behavior of the terminal devices in the smart grid. The results also illustrate the feasibility of monitoring multi-core CPU or multi-type CPU devices using power side-channel information. In future work, we also need to explore the performance of our proposed algorithm on attack code injection, unknown attacks, and long-term attack detection.

## 6. Conclusions

DTU is a key terminal device in the smart power grid, facing more and more threats. However, the traditional detection approaches are not applicable to the DTU. In this paper, we design and implement a lightweight non-intrusive system to detect the tamper of executing programs based on DTU’s power consumption side-channel. We set up a simple testbed of a smart power grid based on DTU in our laboratory and simulate four types of attacks. We chose proper features and compared different ML algorithms for detecting abnormal applications. We show that the detection method achieves a detection accuracy above 99.98% with multiclass classification.

## Figures and Tables

**Table 1:** The confusion matrix for the multiclass classification.

	Normal	Attack1	Attack2	Attack3	Attack4
**Normal**	99.84%(1308)	0.00%(0)	0.14%(2)	0.00%(0)	0.00%(0)
**Attack1**	0.08%(1)	99.77%(1310)	0.15%(2)	0.00%(0)	0.00%(0)
**Attack2**	0.00%(0)	0.00%(0)	100.00%(1341)	0.00%(0)	0.00%(0)
**Attack3**	0.07%(1)	0.00%(0)	0.07%(1)	99.85%(1349)	0.00%(0)
**Attack4**	0.00%(0)	0.00%(0)	0.00%(0)	0.14%(2)	99.86%(1434)

**Table 2:** Classification accuracy and running time of all evaluation programs with different machine learning (ML) models.

Classifier	Total Acc.	Normal	Attack1	Attack2	Attack3	Attack4	Running Time (s)
**GradientBoosting**	99.98%	100.00%	99.91%	100.00%	100.00%	100.00%	0.040716
**DecisionTree**	99.98%	100.00%	99.90%	100.00%	100.00%	100.00%	0.018673
**ExtraTrees**	99.95%	99.81%	99.91%	100.00%	100.00%	100.00%	0.024491
**RandomForest**	99.93%	100.00%	99.91%	100.00%	99.91%	99.83%	0.022847
**GaussianNB**	99.91%	99.81%	100.00%	99.81%	99.91%	100.00%	0.022926
**SVC**	99.89%	100.00%	99.82%	99.81%	99.81%	100.00%	0.196257
**KNeighbors**	99.76%	99.62%	99.73%	99.81%	99.82%	99.83%	3.068105

**Table 3:** The features extracted by pyAudioAnalysis constitute the original feature space.

No.	Feature Name	No.	Feature Name
1	Zero Crossing Rate	7	Spectral Flux
2	Energy	8	Spectral Rolloff
3	Entropy of Energy	9–21	MFCCs
4	Spectral Centroid	22–33	Chroma Vector
5	Spectral Spread	34	Chroma Deviation
6	Spectral Entropy		
